# Numerical solution of a general interval quadratic programming model for portfolio selection

**DOI:** 10.1371/journal.pone.0212913

**Published:** 2019-03-13

**Authors:** Jianjian Wang, Feng He, Xin Shi

**Affiliations:** 1 Donlinks School of Economics and Management, University of Science and Technology Beijing, Beijing, Peoples R China; 2 Business School, Manchester Metropolitan University, Manchester, UK; 3 School of Management, Shanghai University, Shanghai, Peoples R China; 4 Karaganda Medical University, Karaganda, Kazakhstan; Shandong University of Science and Technology, CHINA

## Abstract

Based on the Markowitz mean variance model, this paper discusses the portfolio selection problem in an uncertain environment. To construct a more realistic and optimized model, in this paper, a new general interval quadratic programming model for portfolio selection is established by introducing the linear transaction costs and liquidity of the securities market. Regarding the estimation for the new model, we propose an effective numerical solution method based on the Lagrange theorem and duality theory, which can obtain the effective upper and lower bounds of the objective function of the model. In addition, the proposed method is illustrated with two examples, and the results show that the proposed method is better and more feasible than the commonly used portfolio selection method.

## 1. Introduction

With the mature development of the securities market, in the last decade, studies have paid increasing attention to the theory of portfolio selection. The first quantitative mean variance model for portfolio selection was developed by Markowitz [1], which considers the expected return and variance to be crisp numbers and seeks a balance between two objectives: maximizing the expected return and minimizing the risk in the portfolio selection. Since the 1950s, the quantitative methods for portfolio selection have been dramatically developed in both theories and applications. The deterministic portfolio model that Markowitz developed has been further extended by numerous scholars [2–8]. In these extended portfolio selection models, the coefficients in the objective function and constraint function are always determined as crisp values. However, because of the national economic situation, policy changes, investor psychology and many other factors, the securities market has a strong uncertainty, which causes the dynamic expected returns, risk loss rate and liquidity of the securities market [9]. Moreover, the uncertainties increase the risk of decision-making on portfolio selection for investors. There are two popular approaches to address such uncertainties: (i) fuzzy programming and (ii) interval programming. Since the future returns of each securities cannot be correctly reflected by the historical data, particularly in an uncertain environment, investors can use the fuzzy set to estimate the vagueness of security returns and risk for the future [10–15], which is a good method to address the portfolio selection. The fuzzy programming treats the uncertain quantities as a fuzzy set with certain membership functions. Thus, the decision maker must have precise knowledge of the grade of membership function, which is not easy to obtain from the limited data that the decision maker often has in practice. In fact, another method to address the uncertainty in the portfolio selection problem assumes that the data are not well defined but can vary in given intervals [16]. Hence, interval programming is appropriate to handle the imprecise input data. The existing literatures indicate that interval programming has become a popular topic in the research of portfolio selection because it can enrich the theory of optimization and provide the solution of the problem more practical significance.

At present, the interval programming of portfolio selection is mostly based on the linear format, which is relatively simple compared with non-linear programming. Interval linear programming problems have been explored in several studies on models and estimation methods [17–22]. Then, it has been extensively applied to portfolio selection studies. Based on the interval order relation, Lai et al. (2002) and Lu et al. (2004) proposed an interval programming portfolio selection by quantifying the covariance and expected return as intervals, respectively [23–24]. The difference is that the latter introduces a risk preference coefficient. In solving the multi-objective and multi-period interval portfolio selection optimization model, Giove et al. (2006) proposed the use of a minimax regret approach based on a regret function, and Liu (2013) designed an improved particle swarm optimization algorithm for solution, both of which are used to solve the linear objective function of the interval portfolio model [25–26]. Bhattacharyya et al. (2011) proposed three different mean–variance–skewness models with interval numbers to extend the classical mean-variance portfolio selection model by defining the future financial market optimistically, pessimistically, and weightedly combined ways [27]. Inspired and motivated by [28], Wu et al. (2013) proposed an interval portfolio model, where both expected returns and risk can vary in estimated intervals [29]. In other words, the solution methods to solve the interval linear programming model for portfolio selection have been widely explored. However, to the best of our knowledge, there are few methods to solve the interval quadratic programming model for portfolio selection with interval coefficients of the objective function and its constraints.

Theoretically, robust optimization is also an effective tool for dealing with parameter uncertainty models, and has received extensive attention in the fields of natural sciences, engineering sciences, and economic management. Compared with interval optimization, the robust optimization theory considers the worst case of all possible values, and its optimization result is more conservative than the interval theory. For investors with high security requirements or conservative investment strategies, portfolio strategy based on robust optimization theory is a good choice. However, when using this theory to analyze the problem, if the number of uncertain parameters increases, the number of elements in the scene will also show an exponential growth trend, which makes the established optimization model difficult to solve [30–31]. However, combined with the existing literatures, it is more suitable to use the interval optimization to find the optimal solution of the objective function for the interval quadratic programming portfolio model proposed in this paper [32].

To solve the interval quadratic programming problem, Liu and Wang (2007) developed an algorithm for the interval quadratic programming with constraints, which contained interval numbers [33]. Later, Li and Tian (2008) extended Liu and Wang’s method and developed a new algorithm to optimize the upper bounds of the coefficients in the general interval quadratic programming problem with all coefficients in the objective function, and its constraints are interval numbers [34]. Jiang et al. (2008) conducted a non-linear interval programming method that transformed the uncertain optimization problem into a deterministic two-objective optimization problem to seek the algorithmic solutions [35]. Li et al. (2016) developed a simple and effective method to check the zero dual gaps and discussed some relations between the upper and optimal values of the two modes to estimate the optimal value of the fundamental problem of interval quadratic programming [36]. However, there is little research on the portfolio selection problem using interval quadratic programming. Xu et al. proposed an interval quadratic programming model that assumed that there are no short sales and introduced the acceptability and possibility degree of interval number to transform the uncertainty model into a deterministic model [32,37–38]. Based on a partial-order relation in the set of intervals, Kuamr et al. (2013) developed a method to determine an acceptable optimal feasible solution to solve the generalized interval quadratic programming model, and applied to the securities portfolio selection [39].

Considering transaction cost, borrowing constraint and threshold constraint, Zhang et al. (2016) proposed a multi-stage mean-semi-variance portfolio model with minimum transaction volume constraint [40]. Compared with the existing multi-stage portfolio, the decision variable of the multi-stage portfolio is an integer, which is consistent with the real portfolio. Zhou et al. (2015) constructed a multi-stage portfolio optimization model considering transaction costs. Based on the real frontier, the efficiency of portfolio was defined and the corresponding nonlinear model was proposed to solve the problem [41]. Although Zhang and Zhou considered the transaction volume and transaction cost, it studied the portfolio model of securities under deterministic conditions. However, the various uncertainties in the securities market made it difficult for investors to give accurate values for the yield and risk of securities. Instead, investors were more likely to obtain the range of variation of these uncertain parameters, that is, the number of intervals, so research Investment portfolios and risks were more meaningful for portfolio models with interval numbers. Although Xu et al. (2015) and Kuamr et al. (2013) studied the interval quadratic programming model of securities investment, they did not consider the effects of transaction costs and market liquidity, the results of their proposed models were not sufficiently optimized [32,39]. To construct a more optimized model, a general interval quadratic programming model for portfolio selection based on Xu et al. (2012, 2013) and Kuamr et al. (2013) must be investigated. In this paper, we develop a new general interval quadratic programming model for portfolio selection by introducing the linear transaction costs and liquidity of the securities market, which makes the model more optimized and closer to the actual situation. To solve the general interval quadratic programming, a new solution approach to the problem is proposed based on the Lagrange dual algorithm. Based on the duality method, a more accurate value can be obtained when solving the upper bound of the general interval quadratic programming.

This paper is organized as follows. First, Section 2 reviews some preliminary knowledge about interval numbers. In Section 3, a new general interval quadratic programming model for portfolio selection and a numerical method based on the Lagrange theorem and duality theory are proposed. Then, we present two numerical examples to illustrate the potential applications of the new models and compare two methods of the model in Section 4. Finally, the concluding remarks and future research directions are provided in Section 5.

## 2. Theory of interval numbers

(1) Definition of the interval number and interval matrix

**Definition 2.1** Let $\tilde{a}=[\underline{a},\bar{a}]$ be a bounded closed interval; $\underline{a}\leq \bar{a}$ and $\underline{a},\bar{a}\in R$. We also regard the interval as a number represented by its endpoints $\underline{a}$ and $\bar{a}$. We call $\tilde{a}=[\underline{a},\bar{a}]$ the interval number. If $\underline{a}=\bar{a}$, then $\tilde{a}$ is reduced to a real number.

**Definition 2.2** Let $\tilde{A}={\left({\tilde{a}}_{ij}\right)}_{n\times n}$ be an interval matrix; ${\tilde{a}}_{ij}=\left[{\underline{a}}_{ij},{\bar{a}}_{ij}\right],$. If ${\tilde{a}}_{ij}={\tilde{a}}_{ji}$ and $\tilde{A}$ is positive semidefinite, then we call the interval matrix $\tilde{A}={\left({\tilde{a}}_{ij}\right)}_{n\times n}$ a symmetric positive semidefinite.

(2) Operation of the interval number

Let $\tilde{a}=[\underline{a},\bar{a}]$ and $\tilde{b}=[\underline{b},\bar{b}]$ be two interval numbers and let *k*∈*R* be a real number. Thus, $\tilde{a}+\tilde{b}=[\underline{a}+\underline{b},\bar{a}+\bar{b}]$, $\tilde{a}-\tilde{b}=[\underline{a}-\bar{b},\bar{a}-\underline{b}]$ $\tilde{a}\times \tilde{b}=[min(\underline{a}\cdot \underline{b},\underline{a}\cdot \bar{b},\bar{a}\cdot \underline{b},\bar{a}\cdot \bar{b}),$ $\max(\underline{a}\cdot \underline{b},\underline{a}\cdot \bar{b},\bar{a}\cdot \underline{b},\bar{a}\cdot \bar{b})]$. In particular,

$$k\tilde{a}=\left\{ \begin{array}{l} {}[k\underline{a},k\bar{a}]\;if\;k\geq 0\\ {}[k\bar{a},k\underline{a}]\;if\;k<0 \end{array}\right..$$

For more details on theory of interval numbers, see [42].

## 3. Model and solution

Liu et al. (2015) showed that ignoring transaction costs often leads to invalid portfolio references, so this article introduces the concept of transaction costs [43]. Suppose the investor purchases the risk securities *x*_*i*_(*i* = 1,2,…,*n*) to pay the transaction fee, the rate is *c*_*i*_, and the purchase amount does not exceed the given value *u*_*i*_, the transaction fee is calculated according to *u*_*i*_, then the transaction cost function is defined as follows

$$C_{i}\left(x_{i}\right)=\left\{ \begin{array}{l} 0,x_{i}=0;\\ c_{i}u_{i},0<x_{i}\leq u_{i};\\ c_{i}x_{i},x_{i}>u_{i}. \end{array}\right.$$

When considering the transaction cost, we may set the transaction cost function *C*_*i*_(*x*_*i*_) as a linear function.

This paper introduces the linear transaction costs and liquidity (Following the idea of [9] and[44], this paper suggests using the turnover rate to measure market liquidity) as constraint conditions into the model and uses interval numbers to describe the rate of return, risk loss rate and liquidity of the securities. Suppose there are *n* types of securities for investors to select. Based on the mean-variance model, the investors intend to minimize the risk of the portfolio $f(x)=x^{T}\tilde{Q}x=\sum_{i=1}^{n}\sum_{j=1}^{n}{\tilde{q}}_{ij}x_{i}x_{j}$ under conditions of fixed returns $\sum_{i=1}^{n}{\tilde{R}}_{i}x_{i}-\sum_{i=1}^{n}c_{i}x_{i}\geq {\tilde{R}}_{0}$ and turnover rate $\sum_{i=1}^{n}{\tilde{l}}_{i}x_{i}\geq {\tilde{l}}_{0}$. We establish a new general interval quadratic programming model for portfolio selection as follows:

$$\begin{array}{l} \min f(x)=x^{T}\tilde{Q}x=\sum_{i=1}^{n}\sum_{j=1}^{n}{\tilde{q}}_{ij}x_{i}x_{j}\\ s.t\left\{ \begin{array}{l} \sum_{i=1}^{n}{\tilde{R}}_{i}x_{i}-\sum_{i=1}^{n}c_{i}x_{i}\geq {\tilde{R}}_{0}\\ \sum_{i=1}^{n}{\tilde{l}}_{i}x_{i}\geq {\tilde{l}}_{0}\\ \sum_{i=1}^{n}x_{i}=1\\ x_{i}\geq 0,i=1,2,\cdots ,n \end{array}\right. \end{array}$$ (1)

where *c*_*i*_ is the transaction cost rate of security *i*,*x*_*i*_ is the proportion of security *i*, ${\tilde{r}}_{i}$ is the return of security *i*. ${\tilde{R}}_{i}$ and ${\tilde{l}}_{i}$ denote the expected return and the turnover rate of security *i*, respectively. $\tilde{Q}={\left({\tilde{q}}_{ij}\right)}_{n\times n},i,j=1,2\cdots ,n$ the covariance matrix of the return vector, where we assume that $\tilde{Q}$ is semi-definite. Because ${\tilde{r}}_{i}$, ${\tilde{q}}_{ij}$, and ${\tilde{l}}_{i}$ are uncertain, we treat them as interval numbers, i.e., ${\tilde{r}}_{i}=[{\underline{r}}_{i},{\bar{r}}_{i}]$, ${\tilde{R}}_{i}=E{\tilde{r}}_{i}=[{\underline{R}}_{i},{\bar{R}}_{i}]$, ${\tilde{q}}_{ij}=[{\underline{q}}_{ij},{\bar{q}}_{ij}]$, and ${\tilde{l}}_{i}=[{\underline{l}}_{i},{\bar{l}}_{i}]$. By solving ***x*** = (*x*_1_,*x*_2_,…,*x*_*n*_)^*T*^ in model (1), we obtain a portfolio of securities.

To solve the interval quadratic programming, most studies first consider how to convert it into a deterministic model and design an algorithm [32,45]. Yao et al. (2016) conducted a multi-period mean-variance portfolio selection problem with a stochastic interest rate using the dynamic programming approach and Lagrange duality theory [46]. However, they only considered the expected return and risk in their multi-period mean-variance portfolio selection and did not account for the effects of transaction costs and market liquidity, which makes the result not optimal. This paper focuses on the Lagrange dual algorithm to solve the general interval quadratic programming model for portfolio selection. Based on the duality method, a more accurate value can be obtained when solving for the upper bound of the general interval quadratic programming. Thus, based on the risk range of the portfolio, the investors can select a more reasonable investment plan in an uncertain market environment.

To validate the Lagrange dual method, this paper also uses the common portfolio selection method to solve the general interval quadratic programming model [47]. First, in sections 3.1 and 3.2, this paper proposes a new method based on the Lagrange dual algorithm. Second, conventional method is shown in Section 3.3. Finally, the two methods are compared by experiments.

### 3.1 Decomposition of the model

The objective function and constraint coefficients of model (1) are interval numbers. Clearly, different values of ${\tilde{q}}_{ij},{\tilde{R}}_{i},{\tilde{R}}_{0},{\tilde{l}}_{i}$ and ${\tilde{l}}_{0}$ produce different objective values. Let $S=\{({\tilde{q}}_{ij},{\tilde{R}}_{i},{\tilde{R}}_{0},{\tilde{l}}_{i},{\tilde{l}}_{0})|{\underline{q}}_{ij}\leq {\tilde{q}}_{ij}\leq {\bar{q}}_{ij},{\underline{R}}_{i}\leq {\tilde{R}}_{i}\leq {\bar{R}}_{i},{\underline{R}}_{0}\leq {\tilde{R}}_{0}\leq {\bar{R}}_{0},{\underline{l}}_{i}\leq {\tilde{l}}_{i}\leq {\bar{l}}_{i},{\underline{l}}_{0}\leq {\tilde{l}}_{0}\leq {\bar{l}}_{0},i=1,2,\ldots ,n,j=1,2,\ldots ,n\}$. The values of ${\tilde{q}}_{ij},{\tilde{R}}_{i},{\tilde{R}}_{0},{\tilde{l}}_{i}$ and ${\tilde{l}}_{0}$ can attain the smallest and largest objective value for $f(x)=\sum_{i=1}^{n}\sum_{j=1}^{n}{\tilde{q}}_{ij}x_{i}x_{j}$. Thus, investors can select the appropriate investment options according to the range of the objective function. Based on Li and Tian’s (2008) method [34], model (1) can be transformed into a two-level mathematical programming model (2) and (3). Therefore, we obtain the minimum and maximum values of the objective function by solving (2) and (3), respectively.

$$\begin{array}{l} \underline{f}\left(x\right)=\underset{({\tilde{q}}_{ij},{\tilde{R}}_{i},{\tilde{R}}_{0},{\tilde{l}}_{i},{\tilde{l}}_{0})\in S}{\min}\underset{x}{\min}\sum_{i=1}^{n}\sum_{j=1}^{n}{\tilde{q}}_{ij}x_{i}x_{j}\\ s.t\left\{ \begin{array}{l} \sum_{i=1}^{n}{\tilde{R}}_{i}x_{i}-\sum_{i=1}^{n}c_{i}x_{i}\geq {\tilde{R}}_{0}\\ \sum_{i=1}^{n}{\tilde{l}}_{i}x_{i}\geq {\tilde{l}}_{0}\\ \sum_{i=1}^{n}x_{i}=1\\ x_{i}\geq 0,i=1,2,\cdots ,n \end{array}\right. \end{array}$$ (2)

$$\begin{array}{l} \bar{f}\left(x\right)=\underset{({\tilde{q}}_{ij},{\tilde{R}}_{i},{\tilde{R}}_{0},{\tilde{l}}_{i},{\tilde{l}}_{0})\in S}{\max}\underset{x}{\min}\sum_{i=1}^{n}\sum_{j=1}^{n}{\tilde{q}}_{ij}x_{i}x_{j}\\ s.t\left\{ \begin{array}{l} \sum_{i=1}^{n}{\tilde{R}}_{i}x_{i}-\sum_{i=1}^{n}c_{i}x_{i}\geq {\tilde{R}}_{0}\\ \sum_{i=1}^{n}{\tilde{l}}_{i}x_{i}\geq {\tilde{l}}_{0}\\ \sum_{i=1}^{n}x_{i}=1\\ x_{i}\geq 0,i=1,2,\cdots ,n \end{array}\right. \end{array}$$ (3)

### 3.2 Lagrange dual method to solve the upper and lower bounds

The interval of the objective values of model (1) is obtained by giving its lower bound and upper bound. First, the simpler case to obtain the lower bound is discussed. Since the inner and outer programs of (2) have identical minimization operations, they can be combined into a conventional one-level program, where the constraints of the two programs are simultaneously considered.

For *x*_*i*_,*x*_*j*_≥0(*i*,*j* = 1,2,⋯,*n*), we obtain ${\underline{q}}_{ij}x_{i}x_{j}\leq {\tilde{q}}_{ij}x_{i}x_{j}\leq {\bar{q}}_{ij}x_{i}x_{j}$ In searching for the minimal value of the objective function, parameter ${\tilde{q}}_{ij}(1\leq i,j\leq n)$ must reach its lower bound. Consequently, we have $\underline{f}\left(x\right)=\underset{x}{\min}\sum_{i=1}^{n}\sum_{j=1}^{n}{\underline{q}}_{ij}x_{i}x_{j}$. According to the largest feasible region defined by the inequality constraint in [47]and [48], the constraint inequalities can be transformed into $\sum_{i=1}^{n}{\tilde{R}}_{i}x_{i}-\sum_{i=1}^{n}c_{i}x_{i}\geq {\tilde{R}}_{0}\Rightarrow \sum_{i=1}^{n}({\bar{R}}_{i}-c_{i})x_{i}\geq {\underline{R}}_{0},\sum_{i=1}^{n}{\tilde{l}}_{i}x_{i}\geq {\tilde{l}}_{0}\Rightarrow \sum_{i=1}^{n}{\bar{l}}_{i}x_{i}\geq {\underline{l}}_{0}$. Clearly, model (2) can be written as an equivalent model (4):

$$\begin{array}{l} \underline{f}\left(x\right)=\underset{x}{\min}\sum_{i=1}^{n}\sum_{j=1}^{n}{\underline{q}}_{ij}x_{i}x_{j}\\ s.t.\left\{ \begin{array}{l} \sum_{i=1}^{n}({\bar{R}}_{i}-c_{i})x_{i}\geq {\underline{R}}_{0}\\ \sum_{i=1}^{n}{\bar{l}}_{i}x_{i}\geq {\underline{l}}_{0}\\ \sum_{i=1}^{n}x_{i}=1\\ x_{i}\geq 0,i=1,2,\cdots ,n \end{array}\right. \end{array}$$ (4)

which is a conventional quadratic programming model of portfolio selection.

Now, we consider the upper bound $\bar{f}\left(x\right)$. Note that for *x*_*j*_≥0(*j* = 1,2,⋯,*n*), we have

$$\sum_{i=1}^{n}\sum_{j=1}^{n}{\tilde{q}}_{ij}x_{i}x_{j}\leq \sum_{i=1}^{n}\sum_{j=1}^{n}{\bar{q}}_{ij}x_{i}x_{j}.$$ (5)

So

$$\underset{x}{\min}\sum_{i=1}^{n}\sum_{j=1}^{n}{\tilde{q}}_{ij}x_{i}x_{j}\leq \underset{x}{\min}\sum_{i=1}^{n}\sum_{j=1}^{n}{\bar{q}}_{ij}x_{i}x_{j}.$$ (6)

Hence

$$\underset{({\tilde{q}}_{ij},{\tilde{R}}_{i},{\tilde{R}}_{0},{\tilde{l}}_{i},{\tilde{l}}_{0})\in S}{\max}\underset{x}{\min}\sum_{i=1}^{n}\sum_{j=1}^{n}{\tilde{q}}_{ij}x_{i}x_{j}\leq \underset{({\tilde{q}}_{ij},{\tilde{R}}_{i},{\tilde{R}}_{0},{\tilde{l}}_{i},{\tilde{l}}_{0})\in S}{\max}\underset{x}{\min}\sum_{i=1}^{n}\sum_{j=1}^{n}{\bar{q}}_{ij}x_{i}x_{j}.$$ (7)

However, from ${\bar{q}}_{ij}\in {\tilde{q}}_{ij}(1\leq i,j\leq n)$, we know that

$$\underset{({\tilde{q}}_{ij},{\tilde{R}}_{i},{\tilde{R}}_{0},{\tilde{l}}_{i},{\tilde{l}}_{0})\in S}{\max}\underset{x}{\min}\sum_{i=1}^{n}\sum_{j=1}^{n}{\bar{q}}_{ij}x_{i}x_{j}\leq \underset{({\tilde{q}}_{ij},{\tilde{R}}_{i},{\tilde{R}}_{0},{\tilde{l}}_{i},{\tilde{l}}_{0})\in S}{\max}\underset{x}{\min}\sum_{i=1}^{n}\sum_{j=1}^{n}{\tilde{q}}_{ij}x_{i}x_{j}.$$ (8)

Combining inequalities (7) and (8), we obtain

$$\begin{array}{l} \bar{f}\left(x\right)=\underset{({\tilde{q}}_{ij},{\tilde{R}}_{i},{\tilde{R}}_{0},{\tilde{l}}_{i},{\tilde{l}}_{0})\in S}{\max}\underset{x}{\min}\sum_{i=1}^{n}\sum_{j=1}^{n}{\bar{q}}_{ij}x_{i}x_{j}\\ s.t\left\{ \begin{array}{l} \sum_{i=1}^{n}{\tilde{R}}_{i}x_{i}-\sum_{i=1}^{n}c_{i}x_{i}\geq {\tilde{R}}_{0}\\ \sum_{i=1}^{n}{\tilde{l}}_{i}x_{i}\geq {\tilde{l}}_{0}\\ \sum_{i=1}^{n}x_{i}=1\\ x_{i}\geq 0,i=1,2,\cdots ,n \end{array}\right. \end{array}$$ (9)

because ${\bar{q}}_{ij}\left(i,j=1,2,\ldots ,n\right)$ are real numbers, we denote $S_{1}=\{({\tilde{R}}_{i},{\tilde{R}}_{0},{\tilde{l}}_{i},{\tilde{l}}_{0})|{\underline{R}}_{i}\leq {\tilde{R}}_{i}\leq {\bar{R}}_{i},{\underline{R}}_{0}\leq {\tilde{R}}_{0}\leq {\bar{R}}_{0},{\underline{l}}_{i}\leq {\tilde{l}}_{i}\leq {\bar{l}}_{i},{\underline{l}}_{0}\leq {\tilde{l}}_{0}\leq {\bar{l}}_{0},i=1,2,\ldots ,n,j=1,2,\ldots ,n\}$, and replace the variables as follows: ${\tilde{t}}_{i}=[{\underline{R}}_{i}-c_{i},{\bar{R}}_{i}-c_{i}]$. The upper bound $\bar{f}\left(x\right)$ is formulated as follows:

$$\begin{array}{l} \bar{f}\left(x\right)=\underset{({\tilde{R}}_{i},{\tilde{R}}_{0},{\tilde{l}}_{i},{\tilde{l}}_{0})\in S_{1}}{\max}\underset{x}{\min}\sum_{i=1}^{n}\sum_{j=1}^{n}{\bar{q}}_{ij}x_{i}x_{j}\\ s.t\left\{ \begin{array}{l} \sum_{i=1}^{n}{\tilde{t}}_{i}x_{i}\geq {\tilde{R}}_{0}\\ \sum_{i=1}^{n}{\tilde{l}}_{i}x_{i}\geq {\tilde{l}}_{0}\\ \sum_{i=1}^{n}x_{i}=1\\ x_{i}\geq 0,i=1,2,\cdots ,n \end{array}\right. \end{array}$$ (10)

Solving model (10) is slightly difficult because the outer and inner programs have different directions for optimization (one for maximization and the other for minimization). Now, we compute $\bar{f}\left(x\right)$. We consider the dual form of the inner problem in (10) as follows:

$$\theta (\lambda ,\delta )=\inf\left\{\sum_{i=1}^{n}\sum_{j=1}^{n}{\bar{q}}_{ij}x_{i}x_{j}-\lambda_{1}\left(\sum_{i=1}^{n}{\tilde{t}}_{i}x_{i}-{\tilde{R}}_{0}\right)-\lambda_{2}\left(\sum_{i=1}^{n}{\tilde{l}}_{i}x_{i}-{\tilde{l}}_{0}\right)-\sum_{i=1}^{n}\delta_{i}x_{i}\right\}$$ (11)

where $Q={\left({\tilde{q}}_{ij}\right)}_{n\times n}$ is a symmetric positive semi-definite in model (1). For any *λ*, *δ*, *θ*(*λ*,*δ*) is convex function

The Lagrange dual method on calculating the upper and lower bounds used in Section 3.2 was first proposed by [33]. Then for a special type interval quadratic programming and extended to general interval quadratic programming by [34]. For solving interval quadratic programming (12) with both equality and inequality constraints, algorithms established by [36,49]. So, we can the variable substitution method $r_{1i}=\lambda_{1}{\tilde{t}}_{i}$, $r_{2i}=\lambda_{2}{\tilde{l}}_{i}$ and transform model (11) into model (12) directly by citing [36,49].

$$\begin{array}{l} \bar{f}\left(x\right)=\underset{x,\lambda ,\delta }{\max}(-\sum_{i=1}^{n}\sum_{j=1}^{n}{\bar{q}}_{ij}x_{i}x_{j}+\lambda_{1}{\bar{R}}_{0}+\lambda_{2}{\bar{l}}_{0})\\ s.t\left\{ \begin{array}{l} 2\sum_{j=1}^{n}{\bar{q}}_{ij}x_{j}-r_{1i}-r_{2i}-\delta_{i}=0\\ {\underline{t}}_{i}\lambda_{1}\leq r_{1i}\leq {\bar{t}}_{i}\lambda_{1}\\ {\underline{l}}_{i}\lambda_{2}\leq r_{2i}\leq {\bar{l}}_{i}\lambda_{2}\\ \sum_{i=1}^{n}x_{i}=1\\ \lambda_{1},\lambda_{2}\geq 0,\\ x_{i},\delta_{i}\geq 0,i=1,2,\cdots ,n \end{array}\right. \end{array}$$ (12)

Therefore, the lower bound and upper bound of the objective values $\underline{f}\left(x\right)$ and $\bar{f}\left(x\right)$ are obtained by solving (4) and (12), respectively. Hence, we obtain the intervals of objective functions of the portfolio selection model.

### 3.3 Conventional method to solve the model

To solve the interval programming model, most studies first consider how to convert it into a deterministic model. Many studies converted interval linear programming into deterministic programming in the last decade [50–51]. [47]and [48] introduced definitions such as the best optimal value, worst optimal value, maximum range inequality and minimum range inequality, and they solved the interval linear programming problem by transforming it into deterministic programming. Further, these methods are apply to interval linear programming only, while the portfolio selection model discussed in this paper is a quadratic one. It was proved by [36,49] that these methods can be applied to general interval quadratic programming.

Therefore, for the general interval quadratic programming model (1) of portfolio selection in this paper, we transform the quadratic programming model (1) into two deterministic programming models (13) and (14) directly by using the results in[36,49]. By solving the quadratic programming models (13) and (14), we obtain the upper and lower bounds of the objective function of the general interval quadratic programming model (1) and compare with the results of the proposed Lagrange dual method in this paper. According to the upper and lower bounds of the two methods, we can determine the minimum risk portfolio interval.

$$\begin{array}{l} \min f^{L}(x)=\sum_{i=1}^{n}\sum_{j=1}^{n}{\underline{q}}_{ij}.x_{i}x_{j}\\ s.t\left\{ \begin{array}{l} \sum_{i=1}^{n}{\bar{R}}_{i}x_{i}-\sum_{i=1}^{n}c_{i}x_{i}\geq {\underline{R}}_{0}\\ \sum_{i=1}^{n}{\bar{l}}_{i}x_{i}\geq {\underline{l}}_{0}\\ \sum_{i=1}^{n}x_{i}=1\\ x_{i}\geq 0,i=1,2,\cdots ,n \end{array}\right. \end{array}$$ (13)

$$\begin{array}{l} \min f^{U}(x)=\sum_{i=1}^{n}\sum_{j=1}^{n}{\bar{q}}_{ij}.x_{i}x_{j}\\ s.t\left\{ \begin{array}{l} \sum_{i=1}^{n}{\underline{R}}_{i}x_{i}-\sum_{i=1}^{n}c_{i}x_{i}\geq {\bar{R}}_{0}\\ \sum_{i=1}^{n}{\underline{l}}_{i}x_{i}\geq {\bar{l}}_{0}\\ \sum_{i=1}^{n}x_{i}=1\\ x_{i}\geq 0,i=1,2,\cdots ,n \end{array}\right. \end{array}$$ (14)

## 4. Numerical examples

This section uses two numerical examples to illustrate the proposed method in this paper to solve a general interval quadratic programming model for portfolio selection. We solve the proposed model using the Lagrange dual method (method 1) in this paper and conventional method (method 2) in Section 3.3. To avoid the occasional results of an experiment and ensure the effectiveness of the results, this paper uses two examples to verify.

### 4.1 Example 1

According to [52], we selected three types of securities from April 2005 to March 2009 into Guangzhou Holdings, Shanghai Airport, Minmetals Development. Considering the monthly closing price and turnover rate, we calculated the intervals of expected rate of return, intervals of variance and covariance risk and turnover rate intervals of three securities.

The intervals of expected rate of return are as follows:

$${\tilde{R}}_{1}=[-0.02972,0.02196],{\tilde{R}}_{2}=[-0.02259,0.01803],{\tilde{R}}_{3}=[0.00282,0.06566].$$

The intervals of variance and covariance risk are as follows:

$${\tilde{q}}_{11}=[0.0204,0.0289],{\tilde{q}}_{12}={\tilde{q}}_{21}=[0.0174,0.0212],{\tilde{q}}_{13}={\tilde{q}}_{31}=[0.0213,0.025],$$

$${\tilde{q}}_{22}=[0.0179,0.0269],{\tilde{q}}_{23}={\tilde{q}}_{32}=[0.0164,0.0320],{\tilde{q}}_{33}=[0.0417,0.0590].$$

The turnover rate intervals are as follows:

$${\tilde{l}}_{1}=[0.2724,0.4067],{\tilde{l}}_{2}=[0.2211,0.2569],{\tilde{l}}_{3}=[0.7688,1.2066].$$

Suppose that the transaction costs rates of the three securities are *c*_1_ = 0.00015,*c*_2_ = 0.00025,*c*_3_ = 0.0002. The minimum expected interval return of the three securities was set as ${\tilde{R}}_{0}=[0.001,0.0025]$, i.e., the investors’ expected rate of return is 0.001 in the pessimistic case and is 0.0025 in the optimistic case. The minimum expected turnover rate interval of the three securities was set as ${\tilde{l}}_{0}=[0.40,0.60]$, so 0.40 is the pessimistic case, and 0.60 is the optimistic case.

#### 4.1.1 Solution of method 1

The general interval quadratic programming models (4) and (12) were used to solve the portfolio selection based on Lagrange dual method. By substituting the data of Section 4.1 into models (4) and (12), we obtain

$$\begin{array}{l} \underline{f}(x)=\min(0.0204x_{1}^{2}+0.0348x_{1}x_{2}+0.0426x_{1}x_{3}+0.0179x_{2}^{2}\\ \;+0.0328x_{2}x_{3}+0.0417x_{3}^{2})\\ s.t.\left\{ \begin{array}{l} 0.02196x_{1}+0.01803x_{2}+0.06566x_{3}-\\ (0.00015x_{1}+0.00025x_{2}+0.0002x_{3})\geq 0.001\\ 0.4067x_{1}+0.2569x_{2}+1.2066x_{3}\geq 0.40\\ \sum_{i=1}^{n}x_{i}=1\\ x_{i}\geq 0,i=1,2,3 \end{array}\right. \end{array}$$ (15)

$$\begin{array}{l} \bar{f}\left(x\right)=\max-(0.0289x_{1}^{2}+0.0424x_{1}x_{2}+0.05x_{1}x_{3}+0.0269x_{2}^{2}+0.064x_{2}x_{3}\\ \;+0.059x_{3}^{2})+0.0025\lambda_{1}+0.6\lambda_{2}\\ s.t.\left\{ \begin{array}{l} 2\left(0.0289x_{1}+0.0212x_{2}+0.025x_{3}\right)-r_{11}-r_{21}-\delta_{1}=0\\ 2\left(0.0212x_{1}+0.0269x_{2}+0.032x_{3}\right)-r_{12}-r_{22}-\delta_{2}=0\\ 2\left(0.025x_{1}+0.032x_{2}+0.059x_{3}\right)-r_{13}-r_{23}-\delta_{3}=0\\ -0.02987\lambda_{1}\leq r_{11}\leq 0.02181\lambda_{1}\\ -0.02284\lambda_{1}\leq r_{12}\leq 0.01778\lambda_{1}\\ 0.00262\lambda_{1}\leq r_{13}\leq 0.06546\lambda_{1}\\ 0.2724\lambda_{2}\leq r_{21}\leq 0.4067\lambda_{2}\\ 0.2211\lambda_{2}\leq r_{22}\leq 0.2569\lambda_{2}\\ 0.7688\lambda_{2}\leq r_{23}\leq 1.2066\lambda_{2}\\ \sum_{i=1}^{n}x_{i}=1\\ \lambda_{1},\lambda_{2}\geq 0\\ x_{i},\delta_{i}\geq 0,i=1,2,3 \end{array}\right. \end{array}$$ (16)

Using the function *quadprog* in MATLAB, we derived the optimum solution $\underline{f}\left(x\right),\bar{f}\left(x\right)$. The investment proportions are as follows:

The lower bound of the objective function: $x=(0.0352,0.8197,0.1451),\underline{f}(x)=0.0181.$ The upper bound of the objective function: $x=(0.0188,0.0365,0.9447),\bar{f}(x)=0.0537.$ Combining these results, we conclude that the objective values of this general interval quadratic programming is in the range of *f*(***x***) = [0.0181,0.0537].

#### 4.1.2 Solution of method 2

According to the data in Section 4.1, we obtain the optimal solutions that represent the upper and lower bounds of the objective function of model (1) by solving models (13) and (14) in Section 3.3, respectively. The results are as follows:

The lower bound of objective function is ***x*** = (0.0352,0.8197,0.1451),*f*^*L*^(***x***) = 0.0181.

The upper bound of the objective function is ***x*** = (0,0.0047,0.9953),*f*^*U*^(***x***) = 0.0587.

Then, the solution interval of the portfolio quadratic programming model with transaction costs is *f*(***x***) = [0.0181,0.0587].

#### 4.1.3 Comparison of two methods

The solution intervals for the objective function of the portfolio model obtained using the two methods are *f*_1_ = [0.0181,0.0537] and *f*_2_ = [0.0181,0.0587]. The relationship between the two intervals is shown in S1 Fig.

From the relationship in S1 Fig and interval order relation in [53], we see that *f*_1_⊂*f*_2_. We compare *f*_1_ and *f*_2_ according to the deterministic interval relation (3) in [53]. Since *m*(*f*_1_) = 0.0359<*m*(*f*_2_) = 0.0384, *f*_1_ is better than *f*_2_. Furthermore, *f*_1_ is clearly better than *f*_2_ because *P*(*f*_1_<*f*_2_) = 0.5328, which can be obtained by the interval possibility degree in [51].

In summary, based on the deterministic interval order relation and interval possibility degree, the above results show that the Lagrange dual method of the proposed model in this paper is better than the other method. Moreover, in the actual investment process, according to the method of this paper, the investors can select their preferences based on a specific portfolio plan for forecasting.

### 4.2 Example 2

We selected fifteen types of securities of Shanghai Stock Exchange from September 2006 to September 2018: Pudong Development Bank, Baiyun Airport, Dongfeng Motor, China International Trade, Initial Share, Shanghai Airport, Baogang Stock, Huaneng International, Wantong Expressway, Huaxia Bank, Minsheng Bank, Minmetals Development, Eastern Airlines, SAIC Group, Guangzhou Development. The monthly opening price, closing price and turnover rate of each stock can be obtained from the Wind database, so we can calculate the intervals of expected rate of return, intervals of variance and covariance risk and turnover rate intervals of the fifteen securities as shown in Tables 1–3.

**Table 1:** The intervals of expected rate of return.

**Stock**	**1**	**2**	**3**	**4**	**5**
$\tilde{R}$	[0.0109,0.0221]	[0.0157,0.0224]	[0.0109,0.0236]	[0.0174,0.0259]	[0.0113,0.0276]
**Stock**	**6**	**7**	**8**	**9**	**10**
$\tilde{R}$	[0.0269,0.0340]	[0.0080,0.0236]	[0.0128,0.0205]	[0.0097,0.0204]	[0.0194,0.0300]
**Stock**	**11**	**12**	**13**	**14**	**15**
$\tilde{R}$	[0.0118,0.0224]	[0.0205,0.0414]	[0.0226,0.0390]	[0.0357,0.0480]	[0.0139,0.0243]

**Table 2:** The intervals of variance and covariance risk (Unit /10^−4^).

${\tilde{q}}_{ij}$	1	2	3	4	5	6	7	8	9	10	11	12	13	14	15
**1**	324, 359	138, 153	140, 155	151,167	189,209	156,172	191, 211	131, 144	145, 161	256, 283	256, 283	217, 240	219, 242	220, 243	182, 201
**2**	138, 153	194, 215	157, 173	136, 150	148,163	155, 171	135, 149	112, 124	152, 168	145, 160	119, 131	186, 206	179, 197	160,177	147, 163
**3**	140, 155	157, 173	369, 408	169, 186	199, 220	139, 154	196, 217	156, 172	196, 216	159, 175	119, 132	318, 351	229,253	194,214	198,219
**4**	151, 167	136, 150	169, 186	248,274	158,175	142,157	170,188	113,125	106,117	155,172	151,167	188,208	172,190	173,191	166,183
**5**	189,209	148,163	199,220	158,175	473,523	181,200	231,256	200,221	145,161	207,229	167,185	242,267	230,254	196,217	224,248
**6**	156, 172	155, 171	139, 154	142,157	181,200	205,227	137,151	130,143	132,146	144,159	141,156	182,201	173,192	147,163	152,168
**7**	191, 211	135, 149	196, 217	170,188	231,256	137,151	455,503	161,178	165,183	224,247	186,205	327,361	198,219	224,247	216,238
**8**	131, 144	112, 124	156, 172	113,125	200,221	130,143	161,178	226,249	135,149	164,181	120,132	206,228	194,215	125,138	183,202
**9**	145, 161	152, 168	196, 216	106,117	145,161	132,146	165,183	135,149	312,345	162,180	123,136	215,237	175,193	130,144	182,202
**10**	256, 283	145, 160	159, 175	155,172	207,229	144,159	224,247	164,181	162,180	307,339	244,269	231,256	205,226	208,230	201,222
**11**	256, 283	119, 131	119, 132	151,167	167,185	141,156	186,205	120,132	123,136	244,269	308,340	159,175	154,171	200,221	173,191
**12**	217, 240	186, 206	318, 351	188,208	242,267	182,201	327,361	206,228	215,237	231,256	159,175	608,672	323,357	230,255	238,263
**13**	219, 242	179, 197	229, 253	172,190	230,254	173,192	198,219	194,215	175,193	205,226	154,171	323,357	476,526	186,206	212,234
**14**	220, 243	160, 177	194, 214	173,191	196,217	147,163	224,247	125,138	130,144	208,230	200,221	230,255	186,206	358,396	172,191
**15**	182, 201	147, 163	198, 219	166,183	224,248	152,168	216,238	183,202	182,202	201,222	173,191	238,263	212,234	172,191	302,334

**Table 3:** The turnover rate intervals.

**Stock**	**1**	**2**	**3**	**4**	**5**
$\tilde{l}$	[0.1595,0.1664]	[0.1847,0.1933]	[0.2993,0.3480]	[0.1691,0.1957]	[0.3061,0.3442]
**Stock**	**6**	**7**	**8**	**9**	**10**
$\tilde{l}$	[0.2140,0.2211]	[0.3424,0.3937]	[0.1035,0.1155]	[0.1734,0.1867]	[0.2443,0.2735]
**Stock**	**11**	**12**	**13**	**14**	**15**
$\tilde{l}$	[0.1661,0.1746]	[0.3508,0.3891]	[0.3285,0.3724]	[0.1071,0.1122]	[0.1414,0.1490]

Suppose that the transaction costs rates of the three securities are *c*_*i*_ = 0.0002,(*i* = 1,2,…15). The minimum expected interval return of the three securities was set as ${\tilde{R}}_{0}=[0.0015,0.002]$. The minimum expected turnover rate interval of the three securities was set as ${\tilde{l}}_{0}=[0.05,0.35]$.

#### 4.2.1 Comparison of the results of the two methods

According to the data of Section 4.2, the minimum risk interval and investment ratio of the securities investment portfolio of Example 2 can be obtained by solving models (4) and (12) and models (13) and (14) by MATLAB mathematical software. The results of the two methods are as follows:

Method 1:

The lower bound of the objective function:

$$x=(0,0.2900,0,0.1595,0,0.0912,0,0.2723,0.0772,0,0.1099,0,0,0,0),\underline{f}(x)=0.0147.$$

The upper bound of the objective function:

$$x=(0,0,0.2109,0,0.0885,0.2243,0.2784,0,0,0,0,0.0325,0.1654,0,0),\bar{f}(x)=0.0339.$$

Combining these results, we conclude that the objective values of this general interval quadratic programming is in the range of *f*(***x***) = [0.0147,0.0339].

Method 2:

The lower bound of the objective function:

$$x=(0,0.2900,0,0.1595,0,0.0912,0,0.2723,0.0772,0,0.1099,0,0,0,0),f^{L}(x)=0.0147.$$

The upper bound of the objective function:

$$x=(0,0,0,0,0,0,0.0952,0,0,0,0,0.9048,0,0,0),f^{U}(x)=0.0617.$$

Then, the solution interval of the portfolio quadratic programming model with transaction costs is *f*(***x***) = [0.0147,0.0617].

The solution intervals for the objective function of the portfolio model obtained using the two methods are *f*_1_ = [0.0147,0.0339] and *f*_2_ = [0.0147,0.0617]. The relationship between the two intervals is shown in S2 Fig.

From the relationship shown in S2 Fig and the interval order relation given in[53], we can see that *f*_1_⊂*f*_2_. We compare *f*_1_ and *f*_2_ according to the deterministic interval relation (3) in [53]. Since *m*(*f*_1_) = 0.0243<*m*(*f*_2_) = 0.0382, it can be concluded that *f*_1_ is better than *f*_2_. On the other hand, since *P*(*f*_1_<*f*_2_) = 0.7097, it is clear that *f*_1_ is better than *f*_2_, which can be obtained by the interval possibility degree a in [16].

Therefore, based on the deterministic interval order relation and interval possibility degree, the above results show that the Lagrange dual method of the proposed model in this paper is better than the other method. The results show that smaller interval objective values correspond to a smaller risk of the portfolio. In the actual investment process, according to the method of this paper, the investors can select their preferences based on a specific portfolio plan for forecasting.

## 5. Conclusions

In the actual investment environment, considering the strong uncertainty in the securities market, the paper describes the uncertainties of the securities risk, return and corresponding liquidity with interval numbers and establishes a new general interval quadratic programming model for portfolio selection. Next, we propose a new efficient numerical method to solve the proposed model based on the Lagrange theorem and duality theory. To show the efficiency of the proposed Lagrange dual method, two numerical examples were illustrated. The numerical experiment results show that the proposed portfolio selection model is more feasible, and the Lagrange dual method is better than the traditional method in finding smaller solution intervals, which implies that smaller interval objective values correspond to smaller a risk of the portfolio. In addition, this provides a new investment idea for the securities investors. In the actual securities market, various forms of transaction costs likely affect the portfolio selection. However, this paper only considers the transaction cost as a linear function. There remains considerable research space to solve the quadratic programming model of portfolio selection for different forms of transaction costs.

## Supporting information

S1 Fig Position relation of two interval numbers of Example 1. (TIF)

S2 Fig Position relation of two interval numbers of Example 2. (TIF)

S1 Table The intervals of expected rate of return. (PDF)

S2 Table The intervals of variance and covariance risk (Unit /10^−4^). (PDF)

S3 Table The turnover rate intervals. (PDF)

S1 File Fifteen stock related data sets. (XLSX)
