# Supplementary figures and images for: Numerical solution of a general interval quadratic programming model for portfolio selection
Source: PLoS One. 2019 Mar 13;14(3):e0212913. doi: 10.1371/journal.pone.0212913 (PMC6415890; doi:10.1371/journal.pone.0212913)

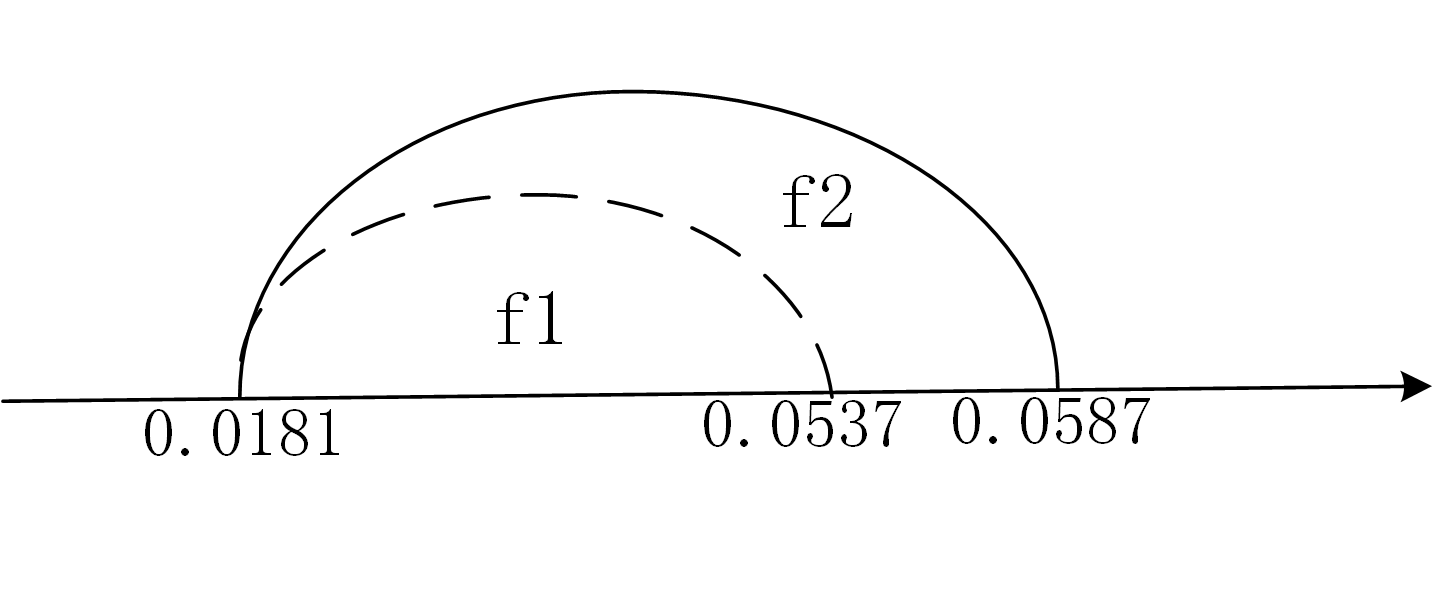

Supplement: S1 Fig — (TIF) [file pone.0212913.s001.tif]

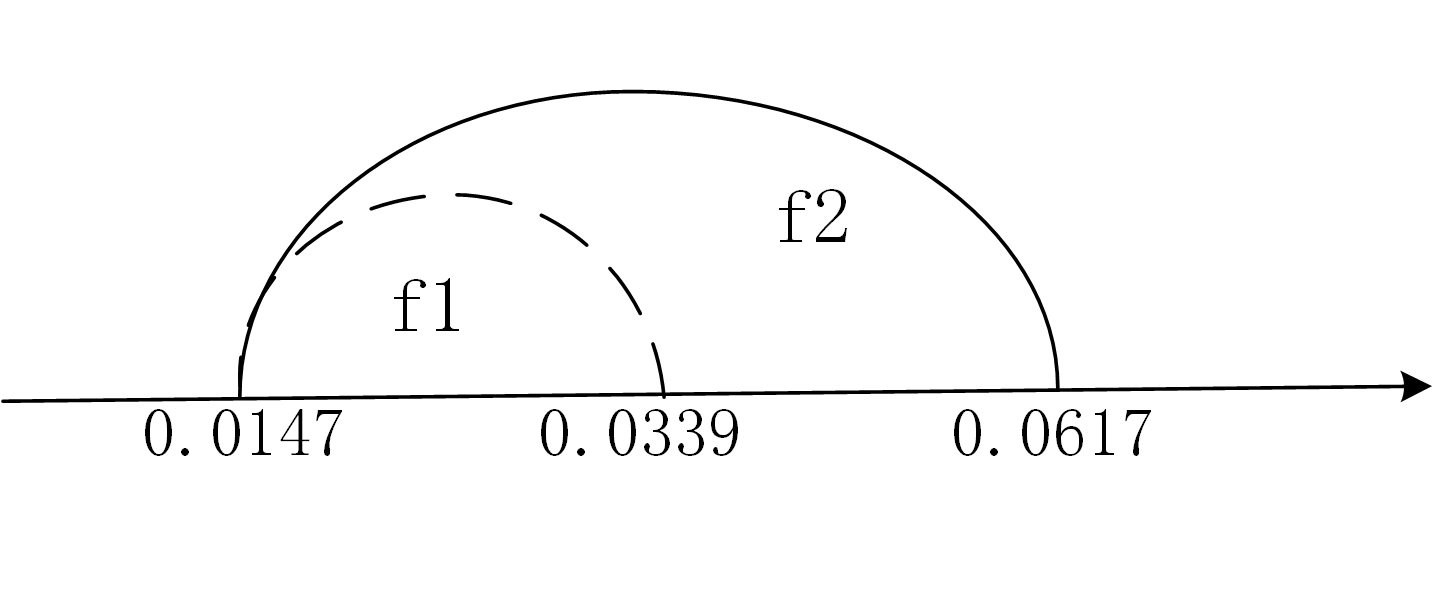

Supplement: S2 Fig — (TIF) [file pone.0212913.s002.tif]
