# Supplementary material for: Numerical solution of a general interval quadratic programming model for portfolio selection
Source: PLoS One. 2019 Mar 13;14(3):e0212913. doi: 10.1371/journal.pone.0212913 (PMC6415890; doi:10.1371/journal.pone.0212913)
Supplement: S1 Table — (PDF) [file pone.0212913.s003.pdf]

S1 Table. The intervals of expected rate of return

**Stock**	**1**	**2**	**3**	**4**	**5**
$\tilde{R}$	[0.0109,0.0221]	[0.0157,0.0224]	[0.0109,0.0236]	[0.0174,0.0259]	[0.0113,0.0276]
**Stock**	**6**	**7**	**8**	**9**	**10**
$\tilde{R}$	[0.0269,0.0340]	[0.0080,0.0236]	[0.0128,0.0205]	[0.0097,0.0204]	[0.0194,0.0300]
**Stock**	**11**	**12**	**13**	**14**	**15**
$\tilde{R}$	[0.0118,0.0224]	[0.0205,0.0414]	[0.0226,0.0390]	[0.0357,0.0480]	[0.0139,0.0243]
