# Supplementary material for: Numerical solution of a general interval quadratic programming model for portfolio selection
Source: PLoS One. 2019 Mar 13;14(3):e0212913. doi: 10.1371/journal.pone.0212913 (PMC6415890; doi:10.1371/journal.pone.0212913)
Supplement: S2 Table — (PDF) [file pone.0212913.s004.pdf]

S2 Table.The intervals of variance and covariance risk (Unit /10-4)

$\tilde{q}_{ij}$	1	2	3	4	5	6	7	8	9	10	11	12	13	14	15
1	324,359	138,153	140,155	151,167	189,209	156,172	191,211	131,144	145,161	256,283	256,283	217,240	219,242	220,243	182,201
2	138,153	194,215	157,173	136,150	148,163	155,171	135,149	112,124	152,168	145,160	119,131	186,206	179,197	160,177	147,163
3	140,155	157,173	369,408	169,186	199,220	139,154	196,217	156,172	196,216	159,175	119,132	318,351	229,253	194,214	198,219
4	151,167	136,150	169,186	248,274	158,175	142,157	170,188	113,125	106,117	155,172	151,167	188,208	172,190	173,191	166,183
5	189,209	148,163	199,220	158,175	473,523	181,200	231,256	200,221	145,161	207,229	167,185	242,267	230,254	196,217	224,248
6	156,172	155,171	139,154	142,157	181,200	205,227	137,151	130,143	132,146	144,159	141,156	182,201	173,192	147,163	152,168
7	191,211	135,149	196,217	170,188	231,256	137,151	455,503	161,178	165,183	224,247	186,205	327,361	198,219	224,247	216,238
8	131,144	112,124	156,172	113,125	200,221	130,143	161,178	226,249	135,149	164,181	120,132	206,228	194,215	125,138	183,202
9	145,161	152,168	196,216	106,117	145,161	132,146	165,183	135,149	312,345	162,180	123,136	215,237	175,193	130,144	182,202
10	256,283	145,160	159,175	155,172	207,229	144,159	224,247	164,181	162,180	307,339	244,269	231,256	205,226	208,230	201,222
11	256,283	119,131	119,132	151,167	167,185	141,156	186,205	120,132	123,136	244,269	308,340	159,175	154,171	200,221	173,191
12	217,240	186,206	318,351	188,208	242,267	182,201	327,361	206,228	215,237	231,256	159,175	608,672	323,357	230,255	238,263
13	219,242	179,197	229,253	172,190	230,254	173,192	198,219	194,215	175,193	205,226	154,171	323,357	476,526	186,206	212,234
14	220,243	160,177	194,214	173,191	196,217	147,163	224,247	125,138	130,144	208,230	200,221	230,255	186,206	358,396	172,191
15	182,201	147,163	198,219	166,183	224,248	152,168	216,238	183,202	182,202	201,222	173,191	238,263	212,234	172,191	302,334
