# Supplementary material for: Numerical solution of a general interval quadratic programming model for portfolio selection
Source: PLoS One. 2019 Mar 13;14(3):e0212913. doi: 10.1371/journal.pone.0212913 (PMC6415890; doi:10.1371/journal.pone.0212913)
Supplement: S3 Table — (PDF) [file pone.0212913.s005.pdf]

S3 Table.The turnover rate intervals

Stock	1	2	3	4	5
$\tilde{l}$	[0.1595,0.1664]	[0.1847,0.1933]	[0.2993,0.3480]	[0.1691,0.1957]	[0.3061,0.3442]
**Stock**	**6**	**7**	**8**	**9**	**10**
$\tilde{l}$	[0.2140,0.2211]	[0.3424,0.3937]	[0.1035,0.1155]	[0.1734,0.1867]	[0.2443,0.2735]
**Stock**	**11**	**12**	**13**	**14**	**15**
$\tilde{l}$	[0.1661,0.1746]	[0.3508,0.3891]	[0.3285,0.3724]	[0.1071,0.1122]	[0.1414,0.1490]
